# Editorial: Current Clinical and Pre-Clinical Progress in Cushing’s Disease

**DOI:** 10.3389/fendo.2020.612321

**Published:** 2020-11-19

**Authors:** Günter Karl Stalla, Eduardo Arzt, Marco Losa, Ulrich Renner

**Affiliations:** ^1^ Medicover Neuroendocrinology, Munich, Germany; ^2^ Instituto de Investigación en Biomedicina de Buenos Aires (IBioBA), Consejo Nacional de Investigaciones Scientificas y Tecnologicas (CONICET), Buenos Aires, Argentina; ^3^ Department of Neurosurgery, San Raffaele Scientific Institute (IRCCS), Milan, Italy; ^4^ Max Planck Institute of Psychiatry (MPI), Munich, Germany

**Keywords:** Cushing’s syndrome, Cushing’s disease, diagnostic score, medical therapy, quality of life, drug targets

Endogenous Cushing’s syndrome (CS) is a rare disease characterized by chronic hypercortisolism. In about 80% of the cases adrenocorticotropin (ACTH) producing tumors (corticotropinomas) of the anterior pituitary are responsible for the hypercortisolism (Cushing’s disease, CD), whereas cortisol-producing adrenal adenomas/carcinomas are responsible for about 15 to 20% of cases of CS. In rare cases, ectopic ACTH-secreting neuroendocrine tumors are the reason for CS. An exotic entity is the so-called factitious CS of which about 20 cases have so far been published worldwide. Pineyro et al. has described such a patient with factitious CS, in which after careful and complex diagnostic procedures finally a psychiatric background of the hypercortisolism was found. Correspondingly the patient’s symptoms of CS disappeared under psychotherapy as it was also observed in most of the previously reported cases of factitious CS.

As the symptoms of hypercortisolism are initially very unspecific, it takes up to 4 years and more until CS is correctly diagnosed. A lot of efforts have been undertaken to shorten the time between onset and diagnosis of CS as this would significantly reduce the mortality and morbidity of affected patients. Braun et al. have suggested a diagnostic score in CS based primarily on clinical signs including osteoporosis, muscular atrophy, red-purple livid striae, plethora, ecchymoses, and dorsocervical fat pat. Patients with these clinical signs should then be biochemically screened using at least two of the three currently reliable tests which are the low-dose dexamethasone suppression test (LDDST), the measurement of 24-h urinary free cortisol (UFC) and the late-night salivary cortisol (LNSC) test. The subsequent diagnostic steps including radiological examinations and measurement of ACTH would then discriminate between ACTH-independent (adrenal tumors) and ACTH-dependent (CD, ectopic ACTH-producing tumors) CS. Concerning the latter, it is known that in particular CD shows a higher prevalence in women as they are 3 to 5 times more often affected than men. In this context, Broersen et al. have studied whether women and men with ACTH-dependent CS present different clinical symptoms. They found that men had a higher prevalence for osteoporosis which persisted after the removal of the ACTH-producing tumors suggesting the need for a careful follow-up of male patients regarding osteoporosis and fractures. However, in general the differences of the clinical patterns between men and women did not justify different sex-specific diagnostic or therapeutic strategies.

Not only the initial diagnosis of CS but also the hypercortisolemia recurrence after surgical removal of ACTH-producing tumors in patients with CD is a diagnostic challenge, as described by Hinojosa-Amaya et al. Currently there is no established definition of CD recurrence and no consensus which tests should be used to confirm recurrence and when these tests should be performed after surgery. Moreover, non-neoplastic hypercortisolemia caused by stress, psychiatric disorders or other co-morbidities may initially mimic recurrence-associated hypercortisolism. To confirm the latter, the LNSC test seems to be the first one to detect elevated cortisol production with both high sensitivity and specificity. Next tests that should follow are the dexamethasone suppression tests (overnight dexamethasone test or LDDST) and UFC. Other biochemical tests may further confirm recurrence but are of limited value. If clinical signs of CD have declined during an initial remission phase the re-appearance of the clinical symptoms is the strongest hint for CD recurrence and should then be confirmed by the previously mentioned biochemical tests.

Regarding therapy, surgical removal of the ACTH producing tumor is the first-line treatment of CD which, however, is not curative in about one third of the patients due to e.g., incomplete tumor resection. For these cases, several medical treatment options are available and new drugs or new formulations of already available drugs are currently tested for safety and efficacy in current clinical trials the stat-of-art of which are summarized in a report of Pivonello et al. Among pituitary-directed drugs pasireotide is the most effective and safe drug if hyperglycemia, its main side effect, is well managed. The long-acting once-monthly intramuscular applied pasireotide-long acting release (LAR) has similar safety and efficacy as the daily applied pasireotide formulation, but shows a better compliance profile. Among adrenal-directed agents, osilodrostat and levoketoconazole show a safe and effective control of CS. Within the group of drugs antagonizing the action of elevated cortisol levels on the glucocorticoid receptor (GR) the non-selective agents mifepristone and relacorilant, which has a better GR-selectivity, improve some of the clinical syndromes and co-morbidities of CS, but have also several side effects. A new approach to treat CD was reported by Ciato and Albani. Based on the fact that in corticotropinomas the negative feedback of cortisol on the ACTH production is impaired, new treatment strategies aim to overcome this glucocorticoid resistance and to restore glucocorticoid sensitivity in corticotroph tumor cells. The mechanism of glucocorticoid resistance is still poorly understood. Recently, two proteins, the heat shock protein 90 (HSP90) a chaperon protein, and testicular receptor 4 (TR4), a nuclear receptor protein have been identified which stabilize and/or mediate the action of GR. The function of both HSP90 and TR4 are impaired in corticotroph tumor cells and there is evidence that restoring their function, in case of HSP90 with the drug silibinin, may also improve the function of the GR.

Due to the multiple metabolic, cardiovascular and neuropsychiatric comorbidities associated with hypercortisolism, patients with active CS have a strongly impaired quality of life (QoL) as reported by Santos et al. After successful treatment and normalization of cortisol levels, patients in remission show an improvement but not a normalization of QoL even at long-term follow up which may in part be a consequence of the long-lasting hypercortisolism before treatment. Education programs for affected patients, support groups, and several other strategies as described by Santos et al. may help improve the patient’s well-being.

In sum, the reports of present Research Topic on Cushing’s syndrome show new developments in establishing a diagnostic score based on a mix of clinical signs and biochemical test which will help to shorten the delay between onset and diagnosis of CS. For the treatment of this disease, new drugs will be available and novel drug targets such as the glucocorticoid receptor have been identified. Moreover, new therapeutic concepts have been initiated to improve the quality of life of patients with Cushing’s disease. However, still a lot of work needs to be done to further improve the clinical management of this disease.

## Author Contributions

All authors contributed to the article and approved the submitted version.

## Funding

Grants to EA: University of Buenos Aires (grant number 20020130100427BA); CONICET (PUE-2016), Agencia Nacional de Promoción Científica y Tecnológica (ANPCyT), Argentina (PICT-2016-1620 and -2018- 03232) and FOCEM-Mercosur (COF 03/11).

## Conflict of Interest

The authors declare that the research was conducted in the absence of any commercial or financial relationships that could be construed as a potential conflict of interest.

